# Improvement in circulating endothelial progenitor cells pool after cardiac resynchronization therapy: increasing the list of benefits

**DOI:** 10.1186/s13287-020-01713-8

**Published:** 2020-05-24

**Authors:** Gonçalo Cristóvão, James Milner, Pedro Sousa, Miguel Ventura, João Cristóvão, Luís Elvas, Artur Paiva, Lino Gonçalves, Carlos Fontes Ribeiro, Natália António

**Affiliations:** 1 Cardiology Department, Coimbra Hospital and University Centre, Coimbra, Portugal; 2 Faculty of Medicine, University of Coimbra, Coimbra, Portugal; 3 Cytometry Operational Management Unit, Clinical Pathology Service, Coimbra Hospital and University Centre, Coimbra, Portugal; 4 Polytechnic Institute of Coimbra, ESTESC-Coimbra Health School, Department Biomedical Laboratory Sciences, Coimbra, Portugal; 5 Clinical Academic Center of Coimbra, Coimbra, Portugal; 6 Coimbra Institute for Clinical and Biomedical Research (iCBR), Coimbra, Portugal

**Keywords:** Endothelial progenitor cells, Cardiac resynchronization therapy, Heart failure, Prognosis

## Abstract

**Background:**

Recent studies suggest that circulating endothelial progenitor cells (EPCs) may influence the response to cardiac resynchronization therapy (CRT). The aim of this study was to evaluate the effect of CRT on EPC levels and to assess the impact of EPCs on long-term clinical outcomes.

**Population and methods:**

Prospective study of 50 patients submitted to CRT. Two populations of circulating EPCs were quantified previously to CRT implantation: CD34^+^KDR^+^ and CD133^+^KDR^+^ cells. EPC levels were reassessed 6 months after CRT. Endpoints during the long-term follow-up were all-cause mortality, heart transplantation, and hospitalization for heart failure (HF) management.

**Results:**

The proportion of non-responders to CRT was 42% and tended to be higher in patients with an ischemic vs non-ischemic etiology (64% vs 35%, *p* = 0.098). Patients with ischemic cardiomyopathy (ICM) showed significantly lower CD34^+^KDR^+^ EPC levels when compared to non-ischemic dilated cardiomyopathy patients (DCM) (0.0010 ± 0.0007 vs 0.0030 ± 0.0024 cells/100 leukocytes, *p* = 0.032). There were no significant differences in baseline EPC levels between survivors and non-survivors nor between patients who were rehospitalized for HF management during follow-up or not. At 6-month follow-up, circulating EPC levels were significantly higher than baseline levels (0.0024 ± 0.0023 vs 0.0047 ± 0.0041 CD34^+^KDR^+^ cells/100 leukocytes, *p* = 0.010 and 0.0007 ± 0.0004 vs 0.0016 vs 0.0013 CD133^+^/KDR^+^ cells/100 leukocytes, *p* = 0.007).

**Conclusions:**

Patients with ICM showed significantly lower levels of circulating EPCs when compared to their counterparts. CRT seems to improve the pool of endogenously circulating EPCs and reduced baseline EPC levels seem not to influence long-term outcomes after CRT.

**Graphical abstract:**

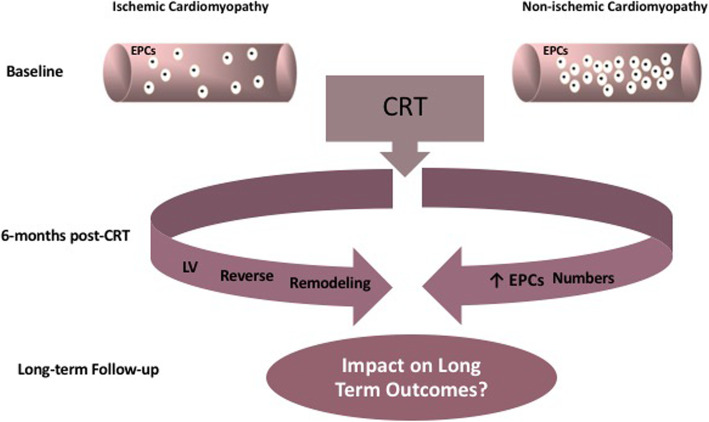

## Introduction

Advanced heart failure (HF) is associated with endothelial dysfunction which negatively impacts cardiac function, heart failure progression, and survival [1, 2]. Circulating endothelial progenitor cells (EPCs) contribute to endothelial homeostasis and may serve as a circulating reservoir for endothelial repair in various pathological conditions [3]. Accumulating evidence shows that reduced circulating EPC levels accurately reflect endothelial dysfunction [4].

In patients with coronary artery disease, reduced EPC levels have been identified as an independent predictor of future cardiovascular events [5, 6]. However, in advanced HF, the association between circulating EPCs and the subsequent long-term clinical outcome remains undefined.

Cardiac resynchronization therapy (CRT) is a well-recognized and important treatment for patients with advanced HF [7]. However, some patients do not respond positively to CRT. Previous studies suggest that endothelial dysfunction may hamper response to CRT [8]. Moreover, previous work by our group suggests that circulating EPC levels may influence CRT response [9]. Nevertheless, no previous studies have specifically focused on the relation of circulating EPCs to subsequent long-term outcomes of advanced HF patients submitted to CRT nor about the effect of CRT on circulating EPCs levels.

The primary objective of this study was to analyze the impact of CRT on circulating EPC pool. As a secondary objective, we intended to assess the potential value of circulating EPCs as a predictor of long-term clinical outcomes after CRT.

## Population and methods

### Study population

This is a prospective study of 50 patients with advanced HF undergoing cardiac resynchronization therapy (CRT) between November 2009 and October 2011 in a single center. Demographic, clinical parameters (including New York Heart Association [NYHA] classification) and echocardiographic parameters of each patient were assessed before and 6 months after CRT. All patients were under stable, optimized medical therapy for CHF at the time of inclusion.

Inclusion criteria were a left ventricular ejection fraction (LVEF) ≤ 35%, QRS ≥ 120 ms with a left bundle branch block morphology, and presence of sinus rhythm.

Exclusion criteria were congenital heart disease, severe valvular disease, acute coronary syndrome or percutaneous coronary intervention within the preceding 3 months, myocardial revascularization surgery or implantation of a previous cardiac pacing device, severe peripheral arterial occlusive disease, anemia (hemoglobin < 8.5 g/dL), renal insufficiency (creatinine > 2.0 mg/dL) or severe noncontrolled arterial hypertension (systolic blood pressure > 180 mmHg or diastolic > 110 mmHg), recent major bleeding requiring blood transfusion (< 6 months), concomitant inflammatory or infectious disease, autoimmune or neoplastic disease, trauma or surgery in the last month, cardiogenic shock, pregnancy, patients taking regular non-steroidal anti-inflammatory drugs or patients taking vasoactive amines or anticoagulants, comorbidities associated with a life expectancy of less than 1 year, and excessive alcohol consumption or illicit drugs abuse.

### Echocardiographic evaluation

Standard echocardiography was performed using Vivid 7 echocardiographs (GE Healthcare, Oslo, Norway) and a 1.7–3.4-MHz tissue harmonic transducer; appropriate software was used (EchoPAC, GE Healthcare). Left ventricular end-systolic volume (LVESV), left ventricular end-diastolic volume (LVEDV), and LVEF were calculated by the biplane Simpson’s equation in apical four-chamber and two-chamber views.

### Long-term follow-up

Data on mortality, heart transplantation, and hospitalization due to worsening heart failure were collected from reviewing hospital records at the closure of the study (April 2018). The echocardiogram performed 6 months after the implantation was used to assess response to CRT. Patients who demonstrated at least a 15% reduction in LVESV at the 6-month follow up were defined as responders to CRT.

### Quantification of circulating EPCs by flow cytometry

Blood samples were collected to evaluate the analytical parameters (including brain natriuretic peptide [BNP]), just before the device implantation. In addition, venous blood samples, stored in ethylenediaminetetraacetic acid (EDTA) tubes, were also collected for quantification of circulating EPC levels and processed within 1 to 2 h after collection. For erythrocyte lysis, FACS Lysing Solution (BD Biosciences) diluted in a ratio of 1:10 (vol/vol) with distilled water was used. Subsequently, a wash with phosphate-buffered saline (PBS) was performed. Hence, 150 μl of whole blood was incubated with 3 antihuman monoclonal antibodies (mAB): 10 μl of APC-conjugated anti-human CD133 mAB (Miltenyi Biotec Inc., Auburn, CA, USA), 10 μl of phycoerythrin (PE) conjugated anti-human KDR mAB (type 2 vascular endothelial growth factor receptor (VEGF-R2)) (Sigma-Aldrich Co., St. Louis, MO, USA), 10 μl of fluorescein isothiocyanate (FITC) conjugated anti-human CD34 mAB (Becton Dickinson and Co.) for 30 min at 4 °C, in the dark. Further flow cytometric analysis was performed on all cases to evaluate for doublets, using a plot of FSC area versus FSC height. The data acquisition was performed in a high-performance flow cytometer, FACSCanto II (BD Biosciences). The Infinicyt 1.7 software (Cytognos, Salamanca, Spain) was used for the analysis. According to the standardized protocol, human circulating EPCs were identified by a minimal antigenic profile that includes at least one marker of immaturity (CD34 and/or CD133), plus at least one marker of endothelial commitment (KDR).

Because EPCs are extremely rare events in peripheral blood, in order to increase the sensitivity of the method and the accuracy of our work, we increased the total number of acquired events to at least 1 million per sample.

Four different populations of angiogenic cells were quantified: CD34^+^, CD133^+^, CD34^+^KDR^+^, and CD133^+^KDR^+^. In the first 30 patients included in the study, these 4 populations were reassessed at 6 months of follow-up.

### Statistical analysis

Statistical analyses were performed using SPSS software version 24 (IBM Corp., Armonk, NY, USA). Continuous variables were tested for normal distribution by Kolmogorov-Smirnov test and expressed as mean ± standard deviation or median ± interquartile range for parametric and nonparametric data, respectively. Categorical data are expressed as counts and percentages. For comparison of continuous data, we used unpaired Student *t* test or nonparametric Mann-Whitney test for variables without a normal distribution. For the comparison of baseline and 6-month follow-up variables, the paired Student *t* test or the Wilcoxon test was used, whichever appropriate. Categorical variables were compared with the chi-square test or with Fisher exact test as appropriate. Kaplan-Meier survival curves were used to evaluate the impact of EPCs levels on time-dependent clinical outcomes. Differences between pairs of survival curves were tested by the log-rank test.

The relationship between variables was calculated using Pearson’s or Spearman’s correlation coefficient, whichever appropriate. A two-tailed *p* value of < 0.05 was considered statistically significant.

## Results

### Baseline characteristics

The baseline characteristics of the study population are presented in Table 1. Among the 50 patients with advanced HF, 11 patients (22%) had an ischemic and 39 a non-ischemic etiology. Mean age was 61.7 ± 10.5 years and the majority of patients were male (64.0 ± 48.5%). Seventy-seven percent of the patients were in NYHA class III, 10.6% in class II, and 12.8% in ambulatory class IV before CRT. The global population had a LVEF of 23.3 ± 6.8%, a heart rate of 70.2 ± 14.6 beats/min, and a QRS duration of 143.4 ± 29.0 ms.

**Table 1:** Baseline characteristics in ischemic and non-ischemic patients

	Ischemic etiology (*n* = 11)	Non-ischemic etiology (*n* = 39)	*p* value
Age (years)^a^	61.5 ± 9.4	61.8 ± 10.9	0.920
Male gender (%)^a^	100.0	53.8	0.004
Years since diagnosis^a^	7.4 ± 5.3	5.8 ± 6.0	0.455
NYHA^a^	2.9 ± 0.3	3.1 ± 0.5	0.390
HR (beats/min)^a^	60.5 ± 7.4	72.8 ± 15.0	0.032
QRS (ms)^a^	130.0 ± 16.3	147.7 ± 31.1	0.093
Diabetes (%)	36.4	18.4	0.209
CKD (%)	10.0	19.4	0.497
Hypertension (%)	55.6	26.5	0.098
Hyperlipidemia (%)	80.0	40.0	0.026
Statin (%)	90.9	50.0	0.016
Acetylsalicylic acid (%)	72.7	21.9	0.002
ACE-inhibitor (%)	72.7	71.9	0.958
AT-1 blocker (%)	9.1	15.6	0.600
Beta-blocker (%)	90.9	87.5	0.768
Spironolactone (%)	45.5	65.6	0.248
Furosemide (%)	90.9	100.0	0.088
Ivabradine (%)	9.1	15.6	0.600
Digoxin (%)	36.4	34.4	0.908
LVESV (mL)^a^	157.7 ± 35.0	200.1 ± 98.5	0.193
LVEDV (mL)^a^	218.3 ± 37.9	250.1 ± 106.2	0.363
LVEF (%)^a^	26.5 ± 6.3	22.3 ± 6.8	0.078
BNP (pg/mL)^a^	381.1 ± 330.5	550.0 ± 602.5	0.458
CRT-D versus CRT-P (%)	100.0/0.0	81.3/18.8	0.308

^a^Mean ± standard deviation

*ACE* angiotensin-converting enzyme, *CKD* chronic kidney disease, *BNP* brain natriuretic peptide, *CRT-D* cardiac resynchronization therapy-defibrillator, *CRT-P* cardiac resynchronization therapy-pacemaker, *HR* heart rate, *LVEDV* left ventricular end-diastolic volume, *LVEF* left ventricular ejection fraction, *LVESV* left ventricular end-systolic volume, *NYHA* New York Heart Association

Regarding the type of device implanted, the proportion of CRT-D and CRT-P was respectively 85.7 and 14.3%. Regarding the chronic medication, 72.1% of the patients were under angiotensin-converting enzyme inhibitors (ACE inhibitors), 88.4% under beta-adrenergic blockers (BB), 60.5% under spironolactone, 97.7% under furosemide, 34.9% under digoxin, 60.5% under statins, 34.9% under aspirin (ASA), and 14.0% under ivabradine. As expected, the proportion of patients treated with statins and ASA was significantly higher in the group of patients with ischemic cardiomyopathy (ICM).

Patients with ICM were more frequently male and had a higher proportion of cardiovascular risk factors (diabetes, hypertension, and hyperlipidemia) than patients with non-ischemic cardiomyopathy (DCM) (Table 1). Moreover, the heart rate was significantly lower in ICM compared to DCM.

Patients with DCM tended to have a lower LVEF value when compared to patients with ICM (22.3 ± 6.8% versus 26.5 ± 6.3%, *p* = 0.078, respectively) (Table 1).

### Circulating EPC levels according to ischemic and non-ischemic etiology

**Fig. 1:**
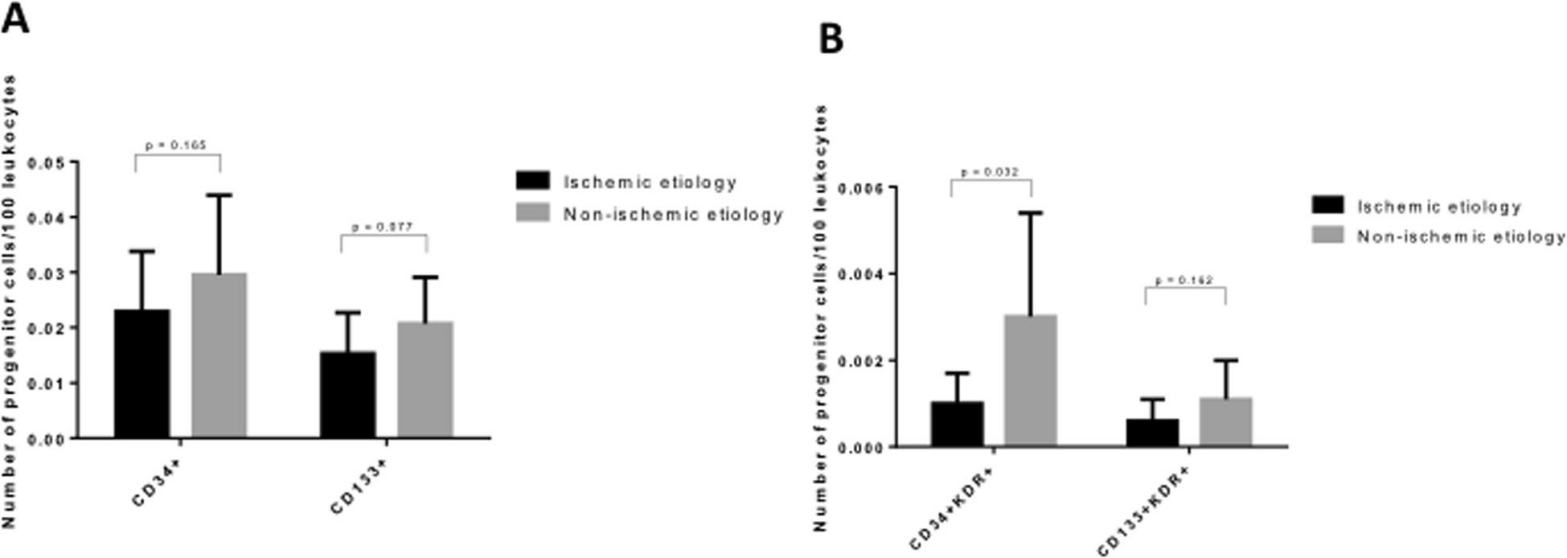
EPCs levels according to ischemic or non-ischemic etiology. **a** Comparison of circulating levels of angiogenic CD34^+^ and CD133^+^ cells between ischemic and non-ischemic patients. **b** Comparison of CD34^+^KDR^+^ and CD133^+^KDR^+^ EPCs levels between ischemic and non-ischemic patients. EPCs, endothelial progenitor cells

There were no statistically significant differences in levels of circulating CD34^+^, CD133^+^, or CD133^+^KDR^+^ cells between ischemic and non-ischemic patients (Fig. 1). However, the CD133^+^ angiogenic cells tended to circulate in lower numbers in patients with ICM compared to patients with an DCM (Fig. 1a). Levels of circulating CD34^+^KDR^+^ EPCs were significantly lower in patients with ICM (Fig. 1b).

### Long-term outcome after CRT

At 6-month follow-up, we observed a significant improvement in LVEF (from 23.7 ± 6.8 to 31.5 ± 11.0%, *p* < 0.001), with a significant decrease in left ventricular volumes (from 189.0 ± 89.7 to 156.4 ± 96.5 ml in LVESV, *p* = 0.004). However, 42% of patients did not respond favorably to CRT according to remodeling criteria.

The proportion of non-responders to CRT tended to be higher in patients with an ischemic etiology by comparison with non-ischemic patients (64% versus 36%, *p* = 0.098) (Table 2).

**Table 2:** Comparison of clinical evolution between ischemic and non-ischemic patients

	Ischemic etiology (*n* = 11)	Non-ischemic etiology (*n* = 39)	*p* value
Number of hospitalizations	1.8 ± 2.0	0.8 ± 1.3	0.052
Rehospitalization for HF (%)	63.6	38.5	0.137
Time until first release (months)	46.8 ± 40.1	53.1 ± 35.4	0.429
CV death (%)	36.4	35.9	0.977
Heart transplantation (%)	9.1	2.6	0.329
Responders (%)	36.4	64.7	0.098

Regarding long-term clinical outcome (mean follow-up of 5.4 ± 2.3 years), 18 patients died: 5/29 (17%) in the responder group and 13/21 (61%) in the non-responder group (*p* = 0.019). Two patients underwent heart transplantation (one responder and one non-responder) and 22 patients were re-hospitalized due to HF: 8/29 (28%) in responder group and 14/21 (67%) in non-responders to CRT (*p* = 0.039).

During follow-up, there were no statistically significant differences in mortality rate or heart transplantation rate between ischemic and non-ischemic patients (supplementary data). However, patients with ICM tended to be more often hospitalized due to HF than DCM patients (mean number of hospitalizations: 1.8 ± 2.0 vs 0.8 ± 1.3, *p* = 0.052, respectively, and hospitalization rate: 63.6% vs 38.5%, *p* = 0.137, respectively) (Table 2).

There were no significant differences in baseline EPC levels among patients who were alive and patients who died during long-term follow-up nor between patients who were rehospitalized for heart failure management or not (supplementary data). Additionally, there was no correlation between baseline EPC levels and time to rehospitalization, number of rehosts or time to death, and survival curves for mortality and rehospitalization due to HF were not significantly different between patients with EPCs numbers under or above the media (supplementary data).

### Evolution of EPC levels after CRT

**Fig. 2:**
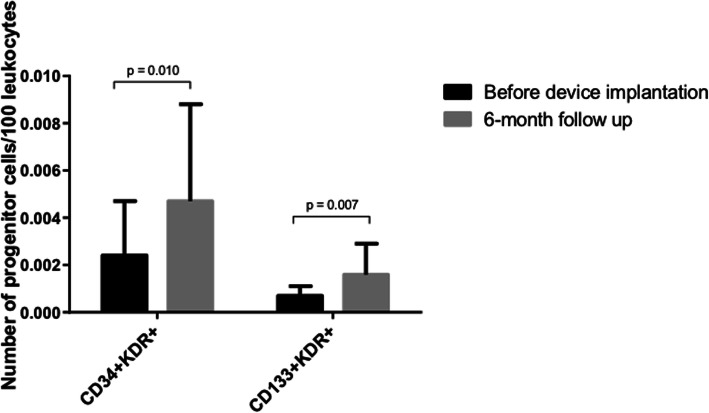
Evolution of EPCs levels from baseline to 6 months after implantation

Six months after, CRT patients presented significantly higher levels of both CD34^+^KDR^+^ and CD133^+^KDR^+^ EPCs than before the implantation (Fig. 2). However, we did not find significant differences in the degree of increase in EPCs between responders and non-responders to CRT.

## Discussion and conclusions

### Discussion

To the best of our knowledge, this is the first study assessing the impact of CRT on circulating EPCs levels.

The main findings of the present work can be summarized as follows. First, the etiology of heart failure seems to influence EPCs levels, with lower number of circulating EPCs in ischemic patients. Second, circulating levels of CD34^+^KDR^+^ and CD133^+^KDR^+^ cells significantly increase after CRT, independently of the patient’s response. Third, baseline EPC numbers seem not to correlate with long-term outcome after CRT.

Heart failure with reduced ejection fraction (HFrEF) is a very common disease with a poor prognosis. The prevalence of HF can be estimated at approximately 1–2% of the adult population in developed countries and the incidence approaches 5–10 per 1000 persons per year [10].

Over the last two decades, CRT has revolutionized the treatment of selected patients who have HFrEF. CRT improves cardiac performance in appropriately selected patients and reduces morbidity and mortality [11]. Several studies have demonstrated the efficacy of CRT in counteracting ventricular remodeling through the recovery of synchronous muscle contractility [12]. However, the exact mechanisms leading to the long-term benefits of CRT are not yet fully understood and other mechanisms beyond left ventricular reverse remodeling are likely involved, explaining the discordance frequently observed between clinical and remodeling response to CRT and also between CRT response and long-term outcomes [13].

End-stage HF is the final common pathway for several different diseases, with ischemic etiology being responsible for the vast majority of cases in developed countries [14].

Previous studies have suggested that patients with ischemic etiology have a lower probability of response to CRT than non-ischemic patients [8, 15]. The reasons for the lack of response to CRT are not well understood. In ischemic etiology, LV desynchrony may be related to segmental wall motion abnormalities due to the presence of myocardial scars or perfusion defects that cannot be resynchronized [15]. Here, we verify that patients with ICM express significantly lower levels of circulating EPCs, suggesting that this pauperization may justify why ICM patients typically benefit less from CRT. However, several studies conducted in recent years have found that the benefits of CRT appear to be similar in HF regardless of the underlying cause. Therefore, presently, the decision to indicate CRT is not influenced by the etiology of HF [14].

Endothelial dysfunction has been extensively reported in patients with HF [16]. Endothelial damage or ischemia leads to the liberation of several mediators, such as VEGF, stromal cell-derived factor 1 (SDF-1), or nitric oxide synthase (NOS). This cascade activation seems to stimulate the proliferation of EPCs in bone marrow and their release to the bloodstream. Circulating EPCs adhere to the injured endothelium, playing a crucial role in vascular repair [17]. During recent years, accumulating evidence revealed that circulating EPCs showed reduced numbers and functional impairment within several cardiovascular diseases. Valgimigli et al. were the first to evaluate the role of circulating EPCs in HF patients. They showed decreasing EPC levels with more advanced stages of congestive HF indicated by higher NYHA classes and elevated NT-proBNP levels [18]. Nonaka-Sarukawa et al. also showed that HF patients present lower EPCs counts than controls [2]. The reduction of circulating EPCs levels in advanced HF can be justified by diffuse and severe endothelial damage. However, conflicting results about the behavior of circulating EPCs in advanced HF have been published. Theiss et al. found that circulating EPCs were lower in patients with ICM than DCM but still higher than healthy controls [19]. Heeschen et al. observed a functional impairment of bone marrow-derived EPCs leading to a reduced migratory capacity into the circulation of patients with ischemic HF compared to healthy controls [20]. However, findings from other investigators groups indicate that the etiology of HF does not differentially affect circulating EPCs [18]. In our study, despite the greater use of statins (a stimulus for EPCs) in patients with ischemic etiology, they showed significantly lower levels of circulating EPCs when compared to non-ischemic patients. This reduced circulating EPCs levels that were observed for both the CD34^+^KDR^+^ cells and for the more immature CD133^+^KDR^+^ population. That difference could potentially explain why ICM patients typically benefit less from CRT and the worse prognosis generally associated with ischemic etiology compared to non-ischemic causes of HF.

#### Long-term outcome after CRT

Low circulating EPC levels are associated with adverse outcomes in patients with coronary artery disease [5]. However, regarding CHF, Michowitz et al. showed that higher levels of EPCs independently predicted all-cause mortality [21]. In contrast, Koller et al. showed that EPCs defined as CD34^+^CD45^dim^KDR^+^ cells were a strong and independent inverse predictor of mortality in patients with chronic HF [22]. Similarly, Samman Tahhan et al. demonstrated that lower EPC counts were strongly and independently predictive of mortality [23]. On the other hand, another study found that CD34^+^KDR^+^ levels were not related with the risk of mortality, composite outcomes, or hospital admissions in patients with ambulatory left ventricular ejection fraction < 40% [24]. However, the potential impact of circulating EPCs on clinical outcomes after CRT had not yet been studied. In our study, baseline EPC levels were not related with long-term outcomes in HF submitted to CRT.

#### Evolution of EPC levels after CRT

An important observation of our study is that numbers of both EPC populations (CD34^+^KDR^+^ cells and CD133^+^KDR^+^ cells) significantly increase after CRT. We can speculate that this increase in EPCs is a result of effective CRT which may translate in an improved capacity of endothelial repair mediated by EPCs. However, the significance of this finding remains to be determined.

In recent years, the role of EPCs in cardiovascular disease and the interplay between inflammation and endothelial progenitor cell biology have been discussed. In patients at an increased cardiovascular risk (diabetes mellitus, systemic hypertension, and hyperlipidemia), EPCs show a decreased proliferative capacity and present reduced levels in peripheral circulation [6, 25]. In patients with advanced CHF, the majority of studies indicate that circulating EPC levels are profoundly decreased [2, 18, 20].

HF is characterized by a chronic inflammatory status with elevated pro-inflammatory cytokines, such as tumor necrosis factor (TNF)-α, interleukin (IL)-1β, and IL-6. This inflammatory milieu can negatively impact on circulating EPCs [26, 27].

Previous studies have shown that CRT reduces the inflammatory milieu of chronic HF [26, 27]. Theodorakis et al. showed that IL-6 and TNF-α were reduced after 3 months of biventricular pacing [26]. In the present study, circulating EPCs significantly increase after CRT. Therefore, we can speculate that this anti-inflammatory action of CRT can be translated into increase in circulating levels of CD34^+^KDR^+^ and CD133^+^KDR EPCs. However, these findings need confirmation and possible mechanisms to explain this association need further investigation.

#### Limitations

This study had a relatively small sample size, and future larger studies would be important to confirm that circulating EPCs do not influence long-term prognosis of HF patients submitted to CRT.

We were not able to explore other functional characteristics of EPCs that might provide further understanding about the role of CRT on EPC response and its contribution to HF pathogenesis.

### Conclusions

Our study shows that patients with ICM present a pauperization in the EPC pool, and it suggests that CRT may increase circulating EPCs levels. Additionally, reduced baseline EPC numbers seem not to influence long-term outcomes after CRT. However, further studies are warranted to better understand the role of EPCs in advanced HF and its potential relation to the beneficial effects of CRT.

## Supplementary information

**Additional file 1.**

## Data Availability

The datasets used and/or analyzed during the current study are available from the corresponding author on reasonable request.

## Abbreviations

ACE-inhibitors: Angiotensin-converting enzyme inhibitors
ASA: Aspirin
CRT: Cardiac resynchronization therapy
DCM: Dilated cardiomyopathy (non-ischemic cardiomyopathy)
EDTA: Ethylenediaminetetraacetic acid
EPCs: Endothelial progenitor cells
FITC: Fluorescein isothiocyanate
HF: Heart failure
HFrEF: Heart failure with reduced ejection fraction
ICM: Ischemic cardiomyopathy
IL: Interleukin
LVEDV: Left ventricular end-diastolic volume
LVEF: Left ventricular ejection fraction
LVESV: Left ventricular end-systolic volume
NYHA: New York Heart Association
NOS: Nitric oxide synthase
PE: Phycoerythrin
SDF-1: Stromal cell-derived factor 1
TNF: Tumor necrosis factor
VEGF-R2: Type 2 vascular endothelial growth factor receptor
