# Supplementary figures and images for: Improvement in circulating endothelial progenitor cells pool after cardiac resynchronization therapy: increasing the list of benefits
Source: Stem Cell Res Ther. 2020 May 24;11:194. doi: 10.1186/s13287-020-01713-8 (PMC7245793; doi:10.1186/s13287-020-01713-8)

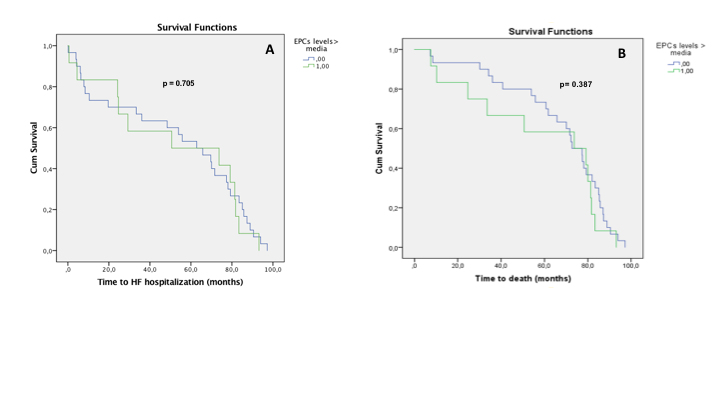

Supplement: Supplementary file 1 — Additional file 1. [file 13287_2020_1713_MOESM1_ESM.tiff]
